# Final diagnoses and mortality rates in ambulance patients administered nebulized β2-agonists bronchodilators

**DOI:** 10.1007/s11739-024-03795-1

**Published:** 2024-11-11

**Authors:** Victor Hagenau, Mathilde G. Mulvad, Jan B. Valentin, Arne S. R. Jensen, Martin F. Gude

**Affiliations:** 1 Department of Research & Development, Prehospital Emergency Medical Services, Central Denmark Region, Aarhus, Denmark; 2 Danish Center for Health Services Research, Department of Clinical Medicine, Aalborg University, Aalborg, Denmark; 3 Department of Clinical Medicine, Aarhus University, Aarhus, Denmark

**Keywords:** Emergency medical services, Lung diseases, Bronchodilator agents, Respiratory insufficiency, Pulmonary Disease, Chronic Obstructive

## Abstract

To assess final diagnoses and mortality rates (30 day and 1 year) in patients treated with the inhaled bronchodilator salbutamol by ambulance personnel, and to establish its role as an identifier of moderate to severe respiratory distress in the prehospital phase of treatment. In a descriptive retrospective observational study, patients experiencing respiratory distress and treated with inhaled bronchodilators, specifically salbutamol, in the prehospital setting within the Central Denmark Region during 2018–2019 were included. The study included 6318 ambulance transports, comprising 3686 cases of acute exacerbation of chronic obstructive pulmonary disease (AECOPD), 234 with community-acquired pneumonia (CAP), 320 with heart disease (HD), 233 adults with asthma, 1674 with various other primary ICD-10 diagnoses (other ≥ 18 years), and 171 patients under 18 years. The 30 day mortality rate for all patients was 10.7% (95% CI 9.8–11.6), with zero deaths within 30 days among adults with asthma and those under 18. Excluding low mortality groups, AECOPD patients had the lowest 30 day mortality at 10.2% (95% CI 9.1–11.3), and HD patients the highest at 15.3% (95% CI 10.6–19.9). The 1-year overall mortality rate increased to 32.1% (95% CI 30.2–34.0), with mortality staying low for asthma and under-18 groups, while differences between other groups lessened and became insignificant. Patients requiring inhaled bronchodilator treatment in ambulances exhibit notably high mortality rates at 30 days and 1 year, except for those with asthma or under 18. The need for prehospital bronchodilators could serve as a clear and unmistakable marker for moderate to severe respiratory distress, enabling early intervention.

**Supplementary Information:**

The online version contains supplementary material available at 10.1007/s11739-024-03795-1.

## Background

Respiratory distress frequently leads to prehospital contact, accounting for 6–12% of total hospital admissions facilitated by the emergency medical service (EMS) [1–6]. Of these patients, up to 40% receive a non-pulmonary diagnosis upon discharge [1, 3], because a multitude of illnesses can result in respiratory distress. Community-acquired pneumonia (CAP), congestive heart failure (CHF), acute exacerbation of chronic obstructive pulmonary disease (AECOPD), and acute asthma are the predominant prehospital conditions associated with this state [3, 7]. Among hospitalized patients, CAP, CHF, and AECOPD collectively exhibit a similar 30-day mortality rate of approximately 10%. To specify, CHF presents a range of 7.8–15% [7–9], AECOPD from 5% to 11.5% [7, 10–13], and for CAP, the range varies from 7 to 13% in hospitalized patients [14–16] and increases to 15.6–27% in intensive care unit (ICU) cases [17, 18]. In contrast, asthma patients transported by ambulance experience exceptionally low mortality [7].

Earlier research has used diverse definitions to characterize respiratory distress in the prehospital setting. At the milder end of this spectrum, this condition has often been identified based on the EMS providers’ impression that the initial EMS contact was for a respiratory-related reason [1, 19]. Notably, in the studies conducted by Prekker et al. and Lindskou et al., only 50% and 63% of the identified patients, respectively, were subsequently admitted to a hospital. Alternatively, respiratory distress has been characterized based on the dispatch code assigned by the Emergency Medical Dispatch Center [2, 4, 5], and it has also been defined only as dyspnea necessitating admission to an Emergency Department [3, 20]. In the study conducted by Pozner et al., eligible patients for inclusion were those who required both hospitalization and treatment for respiratory distress, including medications, advanced airway management, or cardiac monitoring [20]. A method to identify a group of patients with respiratory distress in the prehospital setting might include treatment targeting respiratory difficulties directly such as inhaled bronchodilators. Prehospital treatment with inhaled bronchodilators stimulates β2-receptors in bronchial smooth muscles, promoting airway relaxation and improved airflow, alleviating respiratory distress due to bronchoconstriction [21]. Not all patients experiencing respiratory distress receive treatment with inhaled bronchodilators; the utilization rate for these medications in cases of respiratory distress varies between 20 and 55% [3, 7, 22]. However, in the Central Denmark Region, it is recommended to administer inhaled bronchodilators to all patients having symptoms of prolonged expiration and respiratory distress. Inhaled bronchodilator treatment may serve as an identifier for a homogeneous group of patients with moderate to severe respiratory distress in the prehospital setting—an unambiguously easy identifiable patient cohort not previously examined.

This study aims to determine the final diagnoses and mortality rates (at 30 days and 1 year) among patients treated with inhaled bronchodilators in the prehospital setting and subsequently admitted to the hospital. Additionally, it seeks to establish salbutamol as a clear identifier of patients with moderate to severe respiratory distress in the prehospital phase of treatment.

## Methods

### Study design and setting

This study was a descriptive register-based observational study including patients treated with inhaled bronchodilators in the prehospital setting in the Central Denmark Region in 2018–2019.

This study adhered to the STROBE guidelines (Strengthening the Reporting of Observational Studies in Epidemiology) [23].

### The regional emergency medical service

The region is populated by 1.3 million inhabitants, constituting 23% of the entire Danish population. Within the region there are 8 hospitals differing in size and specialization, with five of them equipped with an emergency department.

The Central Denmark Region has contracted three distinct ambulance services, with a combined workforce of about 650 EMS providers operating 70 ambulances [24, 25]. Ambulances are deployed through the Emergency Medical Dispatch Center (EMDC) when responding to calls received via the national emergency number (112) or calls received directly from the patient’s primary care physician. Each ambulance is staffed by two EMS providers, consisting of either emergency medical technicians or paramedics. Furthermore, the EMDC can dispatch a medical emergency care unit staffed by a prehospital anesthesiologist and a paramedic. Patient transportation primarily prioritizes the nearest hospital, occasionally taking into account the specific medical condition [25].

### Study population

Patients were included in the study if they received treatment with an inhaled β2 bronchodilator medication (specifically salbutamol) and were subsequently admitted to a hospital. The treatment was administered either at the prehospital scene or while being transported by ambulance between January 2018 and December 2019. COVID-19 did not influence EMS management in the study period. We excluded patients with unknown Civil Registration Numbers, and patients that were not admitted to a hospital in the Central Denmark Region. The latter applied to cases where treatment was completed at the scene or when patients were admitted to hospitals located outside the region. Patients were treated with inhaled salbutamol during ambulance transport, with doses ranging from 0.3 mg to 20 mg. The standard initial dose for adult patients, as per the emergency medical service’s standard operating procedure, was 5 mg (equivalent to two doses).

### Exposure

Included patients were divided into six groups based on the main diagnosis established at the hospital. Using the ICD-10 classification system, we identified the following groups: patients with acute exacerbation of chronic obstructive pulmonary disease (AECOPD), community-acquired pneumonia (CAP), any heart-related disease (HD), asthma, patients with another primary ICD-10 (other ≥ 18 years) and patients under the age of 18 (patients < 18 years). A detailed breakdown of the main ICD-10 categories for each group is provided in Supplemental Table S3.

For patients to be classified as having AECOPD, it was necessary for their primary diagnosis to be COPD or for COPD to be listed as a secondary diagnosis alongside a primary diagnosis indicating a lung infection or displaying airway-related symptoms suggestive of a COPD exacerbation (Supplemental Table S3). COPD was defined as the ICD-10 codes J40-J44 [26].

### Outcome

The primary outcome was all-cause 30-day mortality and secondary outcome was all-cause 1 year mortality.

### Data sources

Data from the electronic Prehospital Patient Record (ePPR) utilized by EMS providers were used to identify patients who received inhaled bronchodilator treatment (Salbutamol) during the prehospital phase of care. Vital sign data were also obtained from the ePPR. Prehospital transport data were retrieved from the dispatch system Logis used in the Emergency Medical Dispatch Center. Additionally, data on 30 day and 1 year mortality, length of stay, primary discharge diagnosis, and 10 years of historical diagnoses to determine comorbidity were extracted from the electronic patient record used in hospitals within the Central Denmark Region.

### Statistics

Basal characteristics, vital sign, comorbidities, readmission rate, length of hospital stay and in-hospital treatment were compared between exposure groups as frequencies and percentages or median and interquartile range (IQR) where appropriate. These numbers were presented for all observations as well as unique patients, where in the latter case we used the last observation in chronological order. Comorbidities were categorized according to the Charlson Comorbidity Index based on the ICD-10 diagnostic coding method as specified by Thygesen et al.[27]

Outcomes were presented as incidence rates (IR) and incidence rate ratios (IRR) with 95% confidence intervals (CI) using Poisson regression analysis with the AECOPD group as reference. We used cluster-robust variance to alleviate the assumption of independent observations for patients with multiple observations. In addition, patients with multiple observations were censored at the time of readmission if this occurred within the follow-up period. All analyses were stratified by sex, however, since the exposure group definitions were inherently associated with age and comorbidities, we did not adjust for these. Statistical analyses were performed using Stata 18.0 (StataCorp. 2023. *Stata Statistical Software: Release 18*. College Station, TX: StataCorp LLC.).

## Results

**Fig. 1:**
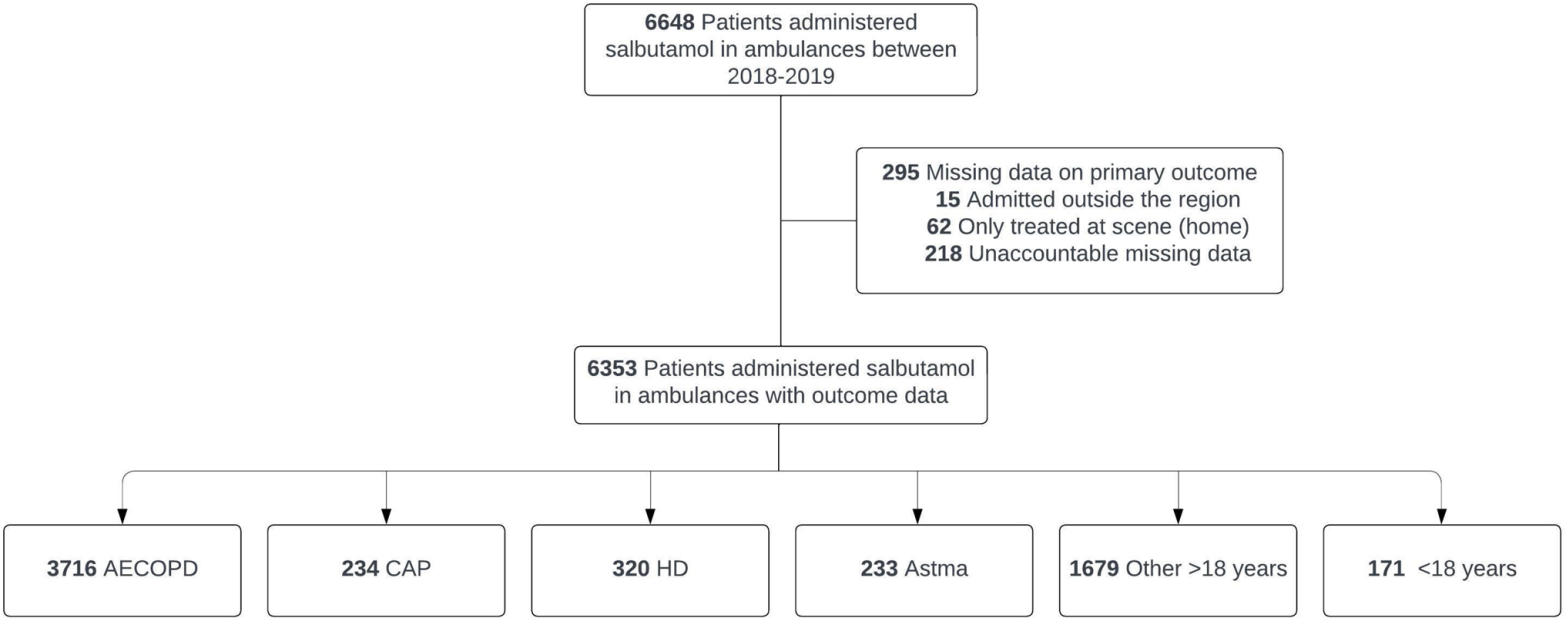
Flowchart. *AECOPD* acute exacerbation of chronic obstructive pulmonary disease, *CAP* Community-acquired pneumonia, *HD* heart disease, *Other* ≥ *18 years* other primary ICD-10 categories

In 2018 and 2019, a total of 4261 patients received prehospital inhaled bronchodilator treatment during 6318 ambulance transports in the Central Denmark Region. The final study cohort was reached after the exclusion of 330 cases lacking primary outcome data, 15 due to hospital admissions outside the region, 62 from home treatment without hospitalization, and 253 having unaccountable missing data (Fig. 1). Within the study cohort, there were 6147 adults (18 years and older) and 171 patients under 18. Among adults, 3686 cases were diagnosed with acute COPD exacerbation, 234 with CAP, 320 with heart disease, and 233 adults with asthma. Additionally, 1674 cases fell under other primary ICD-10 categories (other ≥ 18 years). Moreover, 889 cases had established COPD without acute exacerbation, including 165 in the heart disease group, 718 in the “other ≥ 18 years” group, and 6 in the under-18 age category.

Most patients (75.2%) received the standard initial 5 mg dose of inhaled salbutamol during ambulance transport: 17.1% received 10 mg, 3.2% received 7.5 mg, 2.7% received 2.5 mg, and 1.6% received other doses. Median values across all groups are presented in Table 1.

**Table 1:** Basal characteristics, treatment, hospital admission characteristics and physiological parameters

Factor	AECOPD	CAP	Heart disease	Asthma	Other ≥ 18 years	< 18 years
N	3,686	234	320	233	1,674	171
Sex (female), n (%)	1,944 (52.7%)	144 (61.5%)	171 (53.4%)	162 (69.5%)	875 (52.3%)	73 (42.7%)
Age (years), median (IQR)	74 (66–80)	74 (60–82)	78 (72–84)	45 (26–62)	73 (61–81)	4 (1–11)
Nebulized bronchodilator treatment						
Salbutamol (mg), median (IQR)	5 (5–5)	5 (5–5)	5 (5–5)	5 (5–7.5)	5 (5–5)	2.5 (2.5–5)
Admission characteristics						
Length of hospital stay (days), mean (SD)	1.0 (2.3)	1.1 (2.3)	1.5 (3.1)	0.6 (1.2)	0.7 (2.1)	0.6 (1.1)
ICU-admission, n (%)	40 (1.1%)	3 (1.3%)	13 (4.1%)	1 (0.4%)	13 (0.8%)	2 (1.2%)
Readmission*, n (%)	1,512 (41.0%)	25 (10.7%)	60 (18.8%)	49 (21.0%)	394 (23.5%)	17 (9.9%)
Physiological (vital) parameters (initial assessment in the ambulance)						
Respiratory rate (per minute), median (IQR)	30 (24–33)	28 (24–32)	30 (24–34)	28 (22–30)	28 (24–32)	32 (25–42)
Systolic blood pressure (mmHg), median (IQR)	152 (135–174)	150 (133–170)	155 (131–178.5)	148.5 (131–162.5)	150 (131–172)	123 (112–134)
Diastolic blood pressure (mmHg), median (IQR)	86 (74–100)	85 (74–98)	91.5 (75–109)	88.5 (76–98)	87 (74–100)	77 (65–84)
Heart rate (per minute), median (IQR)	104 (89–117)	105 (88–119)	103 (82–121)	104 (91–118.5)	100 (84–116)	125 (102–143)
GCS (score), median (IQR)	15 (15–15)	15 (15–15)	15 (15–15)	15 (15–15)	15 (15–15)	15 (15–15)
SpO2 < 88, n (%)	1,337 (36.7%)	83 (35.8%)	131 (41.5%)	27 (11.9%)	501 (30.3%)	15 ( 9.3%)
SpO2 88–92%, n (%)	808 (22.2%)	54 (23.3%)	58 (18.4%)	32 (14.1%)	343 (20.8%)	25 (15.4%)
SpO2 93–96%, n (%)	894 (24.6%)	57 (24.6%)	79 (25.0%)	75 (33.0%)	396 (24.0%)	38 (23.5%)
SpO2 97–100%, n (%)	602 (16.5%)	38 (16.4%)	48 (15.2%)	93 (41.0%)	411 (24.9%)	84 (51.9%)

*AECOPD* acute exacerbation of chronic obstructive pulmonary disease, *CAP* Community-acquired pneumonia, *HD* heart disease, *Other* ≥ *18 years* other primary ICD-10 categories, *ICU* intensive care unit, *GCS* Glasgow Coma Scale; *SpO2* Peripheral Capillary Oxygen Saturation measured by pulse oximeter

*Patients with one or more readmissions to the hospital in the study period

Among the 6,318 ambulance transports analyzed, 2,057 cases involved readmissions within the study period, resulting in a readmission rate of 32.6% (95% CI 31.4–33.7). During the two-year study period, the distribution of readmissions was as follows: 588 patients were readmitted once, 228 patients twice, 166 patients between three and five times, 34 patients more than five times, and 11 patients more than ten times. The readmission rates showed no significant difference between sexes (data not shown). However, a significant variation was observed among patient groups. The highest readmission rates were found in patients with AECOPD, while the lowest rates were seen in those with CAP and in the under-18 years category (Table 1).

In all patient groups, except for the under-18 years category, there was a slight to moderate predominance of female participants, ranging from 52.3% to 69.5%. In the under-18 years category, the proportion of females was only 42.7% (Table 1). In terms of age, the AECOPD, CAP, HD, and “other ICD-10 ≥ 18 years” groups displayed a comparable median age, ranging from 73 to 78 years. Participants in the asthma group had a notably lower median age of 45 years, while for patients under 18 years of age, the median age was 4.

The prevalence and distribution of comorbidities showed significant similarities between the AECOPD and the “other ICD-10 ≥ 18 years” group, possibly influenced by a relatively large proportion of known COPD patients in the latter (Table 2). Similarly, comorbidity patterns between the asthma and under-18 groups were comparably infrequent, with a consistently higher prevalence of comorbidities in the asthma group, likely due to its higher median age. The HD group displayed a distinct comorbidity profile with a notably high prevalence of prior myocardial infarction and congestive heart disease, along with the highest rates of hypertension, diabetes mellitus, renal disease, and atherosclerosis-related comorbidities such as peripheral vascular disease, cerebrovascular disease, and dementia (Table 2). These findings were further correlated with the Charlson Comorbidity Score, revealing that the asthma and under-18 groups exhibited the lowest scores, while the remaining groups demonstrated relatively similar scores, with the highest scores observed in the HD group (Table 2).

**Table 2:** 10-year comorbidity

Factor	AECOPD	CAP	Heart Disease	Asthma	Other ≥ 18 years	< 18 years
N	3,686	234	320	233	1,674	171
Myocardial infarction	290 (7.9%)	14 ( 6.0%)	56 (17.5%)	4 (1.7%)	117 (7.0%)	0 ( 0.0%)
Congestive heart failure	596 (16.2%)	19 (8.1%)	131 (40.9%)	4 (1.7%)	268 (16.0%)	0 (0.0%)
Peripheral vascular disease	432 (11.7%)	16 (6.8%)	49 (15.3%)	6 (2.6%)	175 (10.5%)	0 (0.0%)
Cerebrovascular disease	469 (12.7%)	31 (13.2%)	47 (14.7%)	5 (2.1%)	232 (13.9%)	1 (0.6%)
Hemiplegia	17 (0.5%)	2 (0.9%)	1 (0.3%)	0 (0.0%)	8 (0.5%)	2 (1.2%)
Dementia	83 (2.3%)	8 (3.4%)	15 (4.7%)	3 (1.3%)	49 (2.9%)	0 (0.0%)
Chronic pulmonary disease *	3,458 (93.8%)	34 (14.5%)	176 (55.0%)	146 (62.7%)	864 (51.6%)	85 (49.7%)
Diabetes mellitus (without complications)	215 (5.8%)	12 (5.1%)	25 (7.8%)	4 (1.7%)	100 (6.0%)	1 (0.6%)
Diabetes mellitus (with chronic complications)	158 (4.3%)	17 (7.3%)	28 (8.8%)	2 (0.9%)	101 (6.0%)	0 (0.0%)
Mild liver disease	63 (1.7%)	3 (1.3%)	2 (0.6%)	0 (0.0%)	40 (2.4%)	0 (0.0%)
Moderate/severe liver disease	14 (0.4%)	3 (1.3%)	0 (0.0%)	0 (0.0%)	14 (0.8%)	0 (0.0%)
Connective tissue disease	148 (4.0%)	13 (5.6%)	18 (5.6%)	12 (5.2%)	77 (4.6%)	1 (0.6%)
Ulcer disease	142 (3.9%)	11 (4.7%)	14 (4.4%)	0 (0.0%)	68 (4.1%)	0 (0.0%)
Moderate/severe renal disease	338 (9.2%)	17 (7.3%)	53 (16.6%)	10 (4.3%)	171 (10.2%)	0 (0.0%)
Any tumor	496 (13.5%)	37 (15.8%)	49 (15.3%)	5 (2.1%)	242 (14.5%)	0 (0.0%)
Leukemia	11 (0.3%)	4 (1.7%)	2 (0.6%)	0 (0.0%)	7 (0.4%)	0 (0.0%)
Lymphoma	44 (1.2%)	1 (0.4%)	2 (0.6%)	1 (0.4%)	17 (1.0%)	0 (0.0%)
Metastatic solid tumor	52 (1.4%)	8 (3.4%)	7 (2.2%)	2 (0.9%)	41 (2.4%)	0 (0.0%)
AIDS	1 (0.0%)	0 (0.0%)	0 (0.0%)	0 (0.0%)	3 (0.2%)	0 (0.0%)
Charlson comorbidities score						
Score 0	3 (0.1%)	18 (7.7%)	0 (0.0%)	46 (19.7%)	126 (7.5%)	84 (49.1%)
Score 1	93 (2.5%)	22 (9.4%)	6 (1.9%)	89 (38.2%)	114 (6.8%)	84 (49.1%)
Score 2	214 (5.8%)	22 (9.4%)	12 (3.8%)	24 (10.3%)	147 (8.8%)	1 (0.6%)
Score 3	543 (14.7%)	30 (12.8%)	30 ( 9.4%)	24 (10.3%)	201 (12.0%)	2 (1.2%)
Score 4	796 (21.6%)	53 (22.6%)	54 (16.9%)	29 (12.4%)	288 (17.2%)	0 (0.0%)
Score 5	714 (19.4%)	34 (14.5%)	60 (18.8%)	14 (6.0%)	286 (17.1%)	0 (0.0%)
Score 6	618 (16.8%)	18 (7.7%)	42 (13.1%)	5 (2.1%)	195 (11.6%)	0 (0.0%)
Score 7	343 (9.3%)	10 (4.3%)	47 (14.7%)	0 (0.0%)	120 (7.2%)	0 (0.0%)
Score 8	170 (4.6%)	11 (4.7%)	29 (9.1%)	0 (0.0%)	89 (5.3%)	0 (0.0%)
Score 9	88 (2.4%)	10 (4.3%)	20 (6.2%)	1 (0.4%)	51 (3.0%)	0 (0.0%)
Score 10	48 (1.3%)	4 (1.7%)	7 (2.2%)	1 (0.4%)	28 (1.7%)	0 (0.0%)
Score 11	30 (0.8%)	1 (0.4%)	10 (3.1%)	0 (0.0%)	17 (1.0%)	0 (0.0%)
Score 12	18 (0.5%)	1 (0.4%)	3 (0.9%)	0 (0.0%)	7 (0.4%)	0 (0.0%)
Score 13	8 (0.2%)	0 (0.0%)	0 (0.0%)	0 (0.0%)	4 (0.2%)	0 (0.0%)
Score 16	0 (0.0%)	0 (0.0%)	0 (0.0%)	0 (0.0%)	1 (0.1%)	0 (0.0%)

All categories displayed as number (n) and %, 10-year Charlson comorbidities based on ICD-10 codes

*AECOPD* acute exacerbation of chronic obstructive pulmonary disease, *CAP* Community-acquired pneumonia, *HD* heart disease, *Other* ≥ *18 years* other primary ICD-10 categories, *AIDS* acquired immunodeficiency syndrome

^*^AECOPD patients excluded from this category received their first diagnosis of COPD during the current admission

In assessing vital signs, comparable values were observed across the groups in terms of respiratory rate, heart rate, systolic, and diastolic blood pressures, except for individuals under the age of 18. This subgroup displayed lower blood pressure and a higher pulse rate, aligning with expected physiological variances in this age category. Notably, oxygen saturation levels exhibited variations among the groups. The asthma group and individuals under 18 demonstrated the highest oxygen saturation levels, whereas the AECOPD, CAP, and HD categories manifested the lowest levels within the study cohort (Table 1).

Exclusively considering unique patient IDs did not alter baseline characteristics, comorbidity, and physiological parameters (supplemental Table S1 andS2).

The length of hospital stay varied little among all groups (0.6–1.5 days). The HD group had the highest proportion of cases admitted to the ICU (Table 1).

Within 30 days of hospital admission, 593 patients in the study population died, resulting in a total mortality rate (IR) of 10.7% (95% CI 9.8–11.6). The 30 day mortality rate in the AECOPD group was 10.2% (95% CI 9.1–11.3), lower than the rates in the CAP, HD, and “other ≥ 18 years” groups, which had 30-day mortality rates of 12.1% (95% CI 7.4–16.8), 15.3% (95% CI 10.6–19.9), and 13.5% (95% CI 11.5–15.4), respectively (Table 3). Notably, in both the asthma and “patients under 18 years” groups, no deaths occurred within 30 days after admission (Table 3).

**Table 3:** Absolute and relative 30-day and 1-year mortality

**Absolute 30-day mortality**	
Groups	IR (95% CI)
AECOPD	0.102 (0.091–0.113)
Community-acquired pneumonia (CAP)	0.121 (0.074–0.168)
Heart Disease (HD)	0.153 (0.106–0.199)
Astma	0.000 (0.000–0.000)
Other ≥ 18 years	0.135 (0.115–0.154)
Patients < 18y	0.000 (0.000–0.000)
Total	0.107 (0.098–0.116)
**Relative 30 day mortality with the AECOPD group as reference**	
Groups	IRR (95% CI)
AECOPD	1 (ref)
Community-acquired pneumonia (CAP)	1.187 (0.792–1.781)
Heart Disease (HD)	1.495 (1.079–2.072)
Astma	n/a
Other ≥ 18 years	1.320 (1.103–1.579)
Patients < 18y	n/a
**Absolute 1-year mortality**	
Groups	IR (95% CI)
AECOPD	0.339 (0.312–0.366)
Community-acquired pneumonia (CAP)	0.343 (0.250–0.436)
Heart disease	0.390 (0.302–0.479)
Astma	0.025 (0.003–0.048)
Other ≥ 18 years	0.368 (0.329–0.406)
Patients < 18y	0.006 (0.000–0.019)
Total	0.321 (0.302–0.340)
**Relative 1-year mortality with the AECOPD group as reference**	
Groups	IRR (95% CI)
AECOPD	1 (ref)
Community-acquired pneumonia (CAP)	1.012 (0.763,1.343)
Heart Disease	1.152 (0.905,1.466)
Astma	0.075 (0.031,0.180)
Other ≥ 18 years	1.085 (0.951,1.238)
Patients < 18y	0.019 (0.003,0.133)

Absolute and relative 30-day and 1-year mortality presented as incidence rates (IR) and incidence rate ratios (IRR) with 95% confidence intervals (CI)

*AECOPD* acute exacerbation of chronic obstructive pulmonary disease, *Other* ≥ *18 years* other primary ICD-10 categories

When conducting a relative comparison of 30 day mortality using AECOPD as the reference, the CAP group exhibited an insignificant 18.7% increase in the mortality rate (IRR 1.187 (95% CI 0.792–1.781)), while the HD and “Other ≥ 18 years” groups demonstrated significant increased rates of 49.5% (IRR 1.495, 95% CI 1.079–2.072) and 32.0% (IRR 1.320, 95% CI 1.103–1.579), respectively (Table 3).

Within 1 year of hospital admission, 1255 patients died, resulting in a total 1-year mortality rate (IR) of 32.1% (95% CI 30.2–34.0). Interestingly, when considering the 1 year mortality rates, the differences between groups diminished to insignificant increased mortality rates ranging from 1.2% to 15.2%, based on IRR between 1.012 (95% CI 0.763–1.343) and 1.152 (95% CI 0.905–1.466) for the CAP, HD, and “Other ≥ 18 years” groups. The 1-year mortality rates remained very low in both the asthma and “patients under 18 years” groups at 2.5% (95% CI 0.3–4.8) and 0.6% (95% CI 0.0–1.9).

The 30-day mortality rates observed among males and females showed differences within the CAP, “Other ≥ 18 years”, and HD groups. In the CAP group, the 30-day mortality rate (IR) for females was 8.9% (95% CI 3.8–14.0), compared with 17.5% (95% CI 8.1–26.9) for males; in the “Other ≥ 18 years” group, the rate was 12.3% (95% CI 9.8–14.8) for females versus 14.7% (95% CI 11.9–17.6) for males. Conversely, in the HD group, females exhibited a higher 30-day mortality rate of 16.8% (95% CI 10.0–23.5) compared to 13.6% (95% CI 7.1–20.0) for males (Table 4).

**Table 4:** Absolute and relative 30-day and 1-year mortality stratified by sex

	Females	Males
Patients (unique ID), n	2293	1968
Ambulance transports, n	3369	2949
Age all groups (years), median (IQR)	73 (63; 80)	73 (63; 80)
Absolute 30 day mortality		
Groups	IR (95% CI) Females	IR (95% CI) Males
AECOPD	0.104 (0.088–0.120)	0.099 (0.083–0.116)
CAP	0.089 (0.038–0.140)	0.175 (0.081–0.269)
Heart disease	0.168 (0.100–0.235)	0.136 (0.071–0.200)
Astma	0.000 (0.000–0.000)	0.000 (0.000–0.000)
Other ≥ 18 years	0.123 (0.098–0.148)	0.147 (0.119–0.176)
Patients < 18y	0.000 (0.000–0.000)	0.000 (0.000–0.000)
Total	0.104 (0.092–0.116)	0.110 (0.097–0.124)
Relative 30 day mortality with AECOPD as reference		
Groups	IRR (95% CI) Females	IRR (95% CI) Males
AECOPD	1 (ref)	1 (ref)
CAP	0.853 (0.473,1.539)	1.763 (1.005–3.092)
Heart Disease	1.608 (1.046,2.471)	1.363 (0.825–2.253)
Astma	n/a	n/a
Other ≥ 18 years	1.179 (0.914,1.521)	1.483 (1.150–1.913)
Patients < 18y	n/a	n/a
Absolute 1 year mortality		
Groups	IR (95% CI) Females	IR (95% CI) Males
AECOPD	0.334 (0.297,0.370)	0.345 (0.305–0.385)
CAP	0.298 (0.190,0.406)	0.420 (0.248–0.593)
Heart disease	0.423 (0.296,0.550)	0.354 (0.230–0.477)
Astma	0.037 (0.005,0.070)	0.000 (0.000–0.000)
Other ≥ 18 years	0.333 (0.282,0.383)	0.407 (0.348–0.466)
Patients < 18y	0.000 (0.000,0.000)	0.011 (0.000–0.031)
Total	0.308 (0.283,0.333)	0.336 (0.308–0.365)
Relative 1 year mortality with AECOPD as reference		
Groups	IRR (95% CI) Females	IRR (95% CI) Males
AECOPD	1 (ref)	1 (ref)
CAP	0.894 (0.612–1.305)	1.218 (0.796–1.866)
Heart disease	1.267 (0.921–1.743)	1.025 (0.709–1.482)
Astma	0.112 (0.046–0.270)	0.000 (0.000–0.000)
Other ≥ 18 years	0.998 (0.828–1.202)	1.179 (0.979–1.421)
Patients < 18y	0.000 (0.000–0.000)	0.031 (0.004–0.217)

Absolute and relative 30-day and 1-year mortality with the AECOPD group as reference presented as incidence rates (IR) and incidence rate ratios (IRR) with 95% confidence intervals (CI)

*AECOPD* acute exacerbation of chronic obstructive pulmonary disease, *CAP* Community-acquired pneumonia, *HD* heart disease, *Other* ≥ *18 years* other primary ICD-10 categories

These sex-based disparities also extended to the 1-year mortality rate. In the CAP group, females had a lower mortality rate of 29.8% (95% CI 19.0–40.6) compared to 42.0% (95% CI 24.8–59.3) for males. Similarly, in the ‘Other ≥ 18 years’ group, the mortality rate for females was 33.3% (95% CI 28.2–38.3) versus 40.7% (95% CI 34.8–46.6) for males. Meanwhile, in the HD group, females still exhibited the highest 1-year mortality rate at 42.3% (95% CI 29.6–55.0), compared to 35.4% (95% CI 23.0–47.7) for males.

## Discussion

This study characterizes patients receiving inhaled bronchodilator treatment during the prehospital phase of care. Individuals requiring prehospital inhaled bronchodilators constitute a complex and critically ill cohort with a diverse range of comorbidities and high mortality rates. The 30-day all-cause mortality observed in this study totaled 10.7%—comparable to findings in other studies on prehospital respiratory distress [1, 2, 7, 19, 28]. A 1 year mortality rate of 32.1% was identified in the total study cohort, a figure also confirmed by Bøtker et al.[2] The 1-year mortality rate showed relatively limited variations between groups (33.9–39.0%), except for adults with asthma and the group of patients under 18 years where very low mortality rates were observed. Except for these two patient groups, this study’s findings underscore that respiratory distress necessitating inhaled bronchodilator treatment in the ambulance represents an exceptionally life-threatening condition.

Although AECOPD is typically associated with high 30-day mortality rates [11, 29, 30], it unexpectedly had the lowest mortality rate among the studied groups once asthma and patients younger than 18 were excluded. The HD group experienced a significantly higher mortality rate, with a 49.5% increase in 30-day mortality compared to the AECOPD group. This disparity may stem from the study’s inclusion criteria, which likely selected more severe HD cases needing bronchodilator treatment for conditions such as pulmonary edema. Additionally, the HD group had a higher incidence of comorbidities than the other groups (AECOPD, CAP, and “Other ≥ 18 years”), as well as the lowest initial oxygen saturations in the ambulance, a factor independently linked to a high mortality rate. [31, 32] Furthermore, HD patients experienced longer hospital stays and were more frequently admitted to the ICU, indicating a greater severity of their condition. However, when focusing on 1 year mortality rates, the differences between groups became less pronounced. Nevertheless, all groups, except for those with asthma and those younger than 18, exhibited extremely high mortality rates, with the HD group at 39.0%. Research has shown that distinguishing between cardiac-triggered dyspnea and lung-triggered dyspnea in the prehospital setting is difficult [20, 33], which might delay targeted treatments, such as diuretics. The potential for inhaled β2-agonist bronchodilators to worsen the condition in patients with heart failure has been debated, though there does not appear to be substantial evidence supporting this concern [34, 35]. However, the effect of inhaled bronchodilator treatment in patients with heart disease, who do not have COPD, remains uncertain and has been subject to debate [36].

Point-of-care ultrasound could help paramedics to perform more accurate diagnostics in the prehospital phase [37, 38].

Two distinct groups were included in this study: adults with asthma and individuals aged 18 years and younger. These groups stand out from the rest of the cohort, even though they present with respiratory distress requiring inhaled bronchodilator treatment. Both the asthma group and those 18 years and younger have significantly fewer comorbidities and the lowest mortality rates. Notably, the asthma group comprises a significant majority of female participants, at 69.5%. This may indicate a higher prevalence and severity of asthma in women, a phenomenon previously documented in literature [39, 40].

Patients in this study were exclusively identified by a clear prehospital marker: the need for inhaled bronchodilator treatments in ambulances, indicating respiratory distress. This approach contrasts with previous studies that utilized various methods to identify prehospital respiratory distress, such as retrospective analysis post-hospital admission [28, 41], dispatch reference work codes from Emergency Medical Dispatch Centers [2], or subjective impressions of respiratory distress by EMS providers [1, 20, 42]. The diversity in identification methods highlights the diagnostic challenges of prehospital respiratory distress [19, 20, 28, 43]. A reliable and straightforward prehospital identifier is essential not only for facilitating timely interventions [7, 11, 44, 45] but also for ensuring accurate identification of patients for research purposes [45, 46]. The need for inhaled bronchodilator treatment serves as an effective prehospital marker for significant respiratory distress. Furthermore, our study categorized patients into the same four primary groups identified in previous studies—heart failure, COPD, community-acquired pneumonia, and mixed diagnoses. This categorization might account for the relatively similar mortality rates observed across studies among patients admitted to hospital [7, 19, 28, 35]. When compared to previously applied definitions of respiratory distress [1, 19], the need for inhaled bronchodilator treatment almost exclusively identifies patients bound for hospital admission. This group of patients is most likely to benefit from early interventions.

The combination of inhaled bronchodilator treatment and point-of-care ultrasound examinations could assist paramedics in assessing the severity and potentially the cause of respiratory distress, enabling rapid and tailored prehospital treatment [7, 11, 44]. However, further research is necessary to evaluate the effectiveness of multimodal approaches in identifying respiratory distress in the prehospital setting.

One limitation of this study on respiratory distress is the exclusive inclusion of patients who required prehospital bronchodilator treatment, potentially introducing selection bias if the goal was to encompass all individuals experiencing respiratory distress. Nonetheless, this criterion also constitutes a strength, as it ensured the easy identification of all participants, specifically including those with moderate to severe distress—individuals for whom early intervention might be particularly beneficial. The retrospective design of the study, combined with the short-acting nature of salbutamol, restricts our capacity to draw causal inferences between prehospital bronchodilator use and mortality. However, our primary objective was to emphasize the role of inhaled salbutamol as a reliable marker for identifying patients with respiratory distress in the prehospital setting and to describe their associated mortality.

Another limitation is the absence of arterial gas measurements at hospital admission, which prevented comparisons of hypercapnia and respiratory acidosis across different patient groups. Despite these limitations, the study benefited from the high-quality data provided by the Danish Prehospital Patient Record (PPJ), which, with its comprehensive civil registration numbers, ensured nearly complete follow-up [47, 48].

In conclusion, patients who require inhaled bronchodilator treatment for respiratory distress while in the ambulance face notably high mortality rates at both 30 days and 1 year, with the exception of adults with asthma and those aged 18 and under. The need for prehospital inhaled bronchodilator treatment could serve as a clear and easily identifiable prehospital marker of severe respiratory distress, allowing for early interventions.

## Supplementary Information

Below is the link to the electronic supplementary material.

Supplementary file1 (DOCX 27 KB)

## Data Availability

The datasets are available through the corresponding author upon reasonable request and permissions according to Danish legislation.

## Abbreviations

AECOPD: Acute exacerbation of chronic obstructive pulmonary disease
CAP: Community-acquired pneumonia
HD: Heart disease
other ≥ 18 years: Various other primary ICD-10 diagnoses
EMS: Emergency medical service
ICU: Intensive care unit
EMDC: Emergency medical dispatch center
ePPR: Prehospital patient record
IR: Incidence rates
IRR: Incidence rate ratios
