# Supplementary material for: Final diagnoses and mortality rates in ambulance patients administered nebulized β2-agonists bronchodilators
Source: Intern Emerg Med. 2024 Nov 11;20(5):1541–51. doi: 10.1007/s11739-024-03795-1 (PMC12331766; doi:10.1007/s11739-024-03795-1)
Supplement: Supplementary file 1 — Supplementary file1 (DOCX 27 KB) [file 11739_2024_3795_MOESM1_ESM.docx]

Supplemental Table S1: Basal characteristics and physiological parameters for each unique patient (capturing only the last contact in the study period)

| Factor | AECOPD | CAP | Heart Disease | Asthma | Other ≥18 years | <18 years |
| --- | --- | --- | --- | --- | --- | --- |
| N | 2,174 | 209 | 260 | 184 | 1,280 | 154 |
| Sex (female) | 1,180 (54.3%) | 126 (60.3%) | 143 (55.0%) | 124 (67.4%) | 660 (51.6%) | 60 (39.0%) |
| Age, median (IQR) | 74 (67-81) | 76 (62-82) | 78.5 (72-84) | 46 (27.5-62.5) | 73 (61-81) | 4 (1-10) |
| Physiological (vital) parameters (initial assessment in the ambulance) | | | | | | |
| Respiratory rate,  median (IQR) | 29 (24-33) | 28 (24-32) | 30 (25-34) | 26 (22-30) | 28 (24-32) | 32 (25-42) |
| Systolic blood pressure,  median (IQR) | 152 (135-174) | 150 (132-169) | 155 (129-179) | 148 (131-161) | 150 (131-172) | 124 (112-133) |
| Diastolic blood pressure, median (IQR) | 86 (73-100) | 85 (74-95) | 92 (74-109) | 89 (76-99) | 86 (73.5-100) | 77 (65-84) |
| Heart rate, median (IQR) | 104 (89-118) | 105 (88-119) | 102 (83-121) | 106 (92-120) | 100 (83-116) | 126 (105-145) |
| GCS, median (IQR) | 15 (15-15) | 15 (15-15) | 15 (15-15) | 15 (15-15) | 15 (15-15) | 15 (15-15) |
| SpO2 <88 | 848 (39.5%) | 73 (35.3%) | 110 (42.8%) | 18 (10.1%) | 385 (30.6%) | 15 (10.3%) |
| SpO2 88-92% | 491 (22.9%) | 53 (25.6%) | 51 (19.8%) | 27 (15.1%) | 269 (21.4%) | 22 (15.1%) |
| SpO2 93-96% | 497 (23.1%) | 50 (24.2%) | 59 (23.0%) | 64 (35.8%) | 293 (23.3%) | 36 (24.7%) |
| SpO2 97-100% | 311 (14.5%) | 31 (15.0%) | 37 (14.4%) | 70 (39.1%) | 312 (24.8%) | 73 (50.0%) |

Table S1: Basal characteristics, hospital admission and physiological parameters AECOPD = acute exacerbation of chronic obstructive pulmonary disease, CAP = Community-acquired pneumonia, HD = heart disease, Other ≥18 years = other primary ICD-10 categories, ICU = intensive care unit, GCS = Glasgow Coma Scale; SpO2 = Peripheral Capillary Oxygen Saturation measured by pulse oximeter.

Supplemental Table S2: Comorbidity for each unique patient (capturing only the last contact in the study period)

| Factor | AECOPD | CAP | Heart Disease | Asthma | Other ≥18 years | <18 years |
| --- | --- | --- | --- | --- | --- | --- |
| N | 2,174 | 209 | 260 | 184 | 1,280 | 154 |
| Myocardial infarction | 152 (7.0%) | 12 (5.7%) | 44 (16.9%) | 2 (1.1%) | 78 (6.1%) | 0 (0.0%) |
| Congestive heart failure | 320 (14.7%) | 17 (8.1%) | 102 (39.2%) | 4 (2.2%) | 189 (14.8%) | 0 (0.0%) |
| Peripheral vascular disease | 247 (11.4%) | 14 (6.7%) | 42 (16.2%) | 6 (3.3%) | 129 (10.1%) | 0 (0.0%) |
| Cerebrovascular disease | 272 (12.5%) | 29 (13.9%) | 37 (14.2%) | 4 (2.2%) | 171 (13.4%) | 1 (0.6%) |
| Hemiplegia | 12 (0.6%) | 1 (0.5%) | 0 (0.0%) | 0 (0.0%) | 6 (0.5%) | 2 (1.3%) |
| Dementia | 64 (2.9%) | 8 (3.8%) | 14 (5.4%) | 3 (1.6%) | 43 (3.4%) | 0 (0.0%) |
| Chronic pulmonary disease | 1,991 (91.6%) | 25 (12.0%) | 124 (47.7%) | 106 (57.6%) | 542 (42.3%) | 72 (46.8%) |
| Diabetes mellitus (without complications) | 120 (5.5%) | 11 (5.3%) | 20 (7.7%) | 3 (1.6%) | 76 (5.9%) | 1 (0.6%) |
| Diabetes mellitus with chronic complications | 101 (4.6%) | 13 (6.2%) | 24 (9.2%) | 2 (1.1%) | 72 (5.6%) | 0 (0.0%) |
| Mild liver disease | 36 (1.7%) | 2 (1.0%) | 1 (0.4%) | 0 (0.0%) | 25 (2.0%) | 0 (0.0%) |
| Moderate/severe liver disease | 6 (0.3%) | 3 (1.4%) | 0 (0.0%) | 0 (0.0%) | 10 (0.8%) | 0 (0.0%) |
| Connective tissue disease | 93 (4.3%) | 11 (5.3%) | 15 (5.8%) | 9 (4.9%) | 51 (4.0%) | 1 (0.6%) |
| Ulcer disease | 89 (4.1%) | 8 (3.8%) | 12 (4.6%) | 0 (0.0%) | 52 (4.1%) | 0 (0.0%) |
| Moderate/severe renal disease | 179 (8.2%) | 14 (6.7%) | 40 (15.4%) | 5 (2.7%) | 123 (9.6%) | 0 (0.0%) |
| Any tumor | 313 (14.4%) | 33 (15.8%) | 35 (13.5%) | 5 (2.7%) | 196 (15.3%) | 0 (0.0%) |
| Leukemia | 7 (0.3%) | 4 (1.9%) | 0 (0.0%) | 0 (0.0%) | 7 (0.5%) | 0 (0.0%) |
| Lymphoma | 29 (1.3%) | 1 (0.5%) | 2 (0.8%) | 1 (0.5%) | 12 (0.9%) | 0 (0.0%) |
| Metastatic solid tumor | 35 (1.6%) | 6 (2.9%) | 5 (1.9%) | 2 (1.1%) | 37 (2.9%) | 0 (0.0%) |
| AIDS | 1 (0.0%) | 0 (0.0%) | 0 (0.0%) | 0 (0.0%) | 3 (0.2%) | 0 (0.0%) |
| Charlson Comorbidities Score | | | | | | |
| Score 0 | 1 ( <0.1%) | 17 (8.1%) | 0 (0.0%) | 40 (21.7%) | 112 (8.8%) | 80 (51.9%) |
| Score 1 | 49 (2.3%) | 20 (9.6%) | 5 (1.9%) | 69 (37.5%) | 94 (7.3%) | 71 (46.1%) |
| Score 2 | 122 (5.6%) | 18 (8.6%) | 10 (3.8%) | 18 (9.8%) | 117 (9.1%) | 1 (0.6%) |
| Score 3 | 317 (14.6%) | 26 (12.4%) | 26 (10.0%) | 16 (8.7%) | 150 (11.7%) | 2 (1.3%) |
| Score 4 | 453 (20.8%) | 50 (23.9%) | 47 (18.1%) | 22 (12.0%) | 210 (16.4%) | 0 (0.0%) |
| Score 5 | 429 (19.7%) | 30 (14.4%) | 50 (19.2%) | 13 (7.1%) | 211 (16.5%) | 0 (0.0%) |
| Score 6 | 363 (16.7%) | 16 (7.7%) | 32 (12.3%) | 4 (2.2%) | 144 (11.2%) | 0 (0.0%) |
| Score 7 | 204 (9.4%) | 9 (4.3%) | 42 (16.2%) | 0 (0.0%) | 91 (7.1%) | 0 (0.0%) |
| Score 8 | 117 (5.4%) | 10 (4.8%) | 20 (7.7%) | 0 (0.0%) | 70 (5.5%) | 0 (0.0%) |
| Score 9 | 47 (2.2%) | 8 (3.8%) | 13 (5.0%) | 1 (0.5%) | 36 (2.8%) | 0 (0.0%) |
| Score 10 | 32 (1.5%) | 4 (1.9%) | 7 (2.7%) | 1 (0.5%) | 24 (1.9%) | 0 (0.0%) |
| Score 11 | 24 (1.1%) | 0 (0.0%) | 6 (2.3%) | 0 (0.0%) | 10 (0.8%) | 0 (0.0%) |
| Score 12 | 11 (0.5%) | 1 (0.5%) | 2 (0.8%) | 0 ( 0.0%) | 7 (0.5%) | 0 (0.0%) |
| Score 13 | 5 (0.2%) | 0 (0.0%) | 0 (0.0%) | 0 (0.0%) | 3 (0.2%) | 0 (0.0%) |
| Score 16 | 0 (0.0%) | 0 (0.0%) | 0 (0.0%) | 0 (0.0%) | 1 (0.1%) | 0 (0.0%) |

Table S2: 10-year Charlson comorbidities based on ICD-10 codes. *AECOPD patients excluded from this category received their first diagnosis of COPD during the current admission.

AECOPD = acute exacerbation of chronic obstructive pulmonary disease, CAP = Community-acquired pneumonia, HD = heart disease, Other ≥18 years = other primary ICD-10 categories. AIDS = Acquired Immunodeficiency Syndrome.

Supplemental Table S3: Patient groups defined by ICD-10 categories

| ICD-10 Category | Frequency (n) | Percent (%) |
| --- | --- | --- |
| Acute exacerbation of chronic obstructive pulmonary disease (AECOPD) | | |
| A40, A41 | 4 | 0.11 |
| J09-J10 | 23 | 0.62 |
| J12 | 1 | 0.03 |
| J13-J16 | 112 | 3.04 |
| J18 | 275 | 7.46 |
| J20 | 2 | 0.05 |
| J22 | 14 | 0.38 |
| J43 | 2 | 0.05 |
| J44 | 2436 | 66.09 |
| J45 | 69 | 1.87 |
| J47 | 2 | 0.05 |
| J96, J98 | 196 | 5.32 |
| R05, R06 | 550 | 14.92 |
| Total | 3686 | 100.00 |
| Community-acquired pneumonia (CAP) | | |
| J09-J10 | 19 | 8.12 |
| J12 | 1 | 0.43 |
| J13-J16 | 52 | 22.22 |
| J18 | 154 | 65.81 |
| J20 | 5 | 2.14 |
| J22 | 3 | 1.28 |
| Total | 234 | 100.00 |
| Heart disease (HD) | | |
| I20 | 8 | 2.50 |
| I21-I24 | 47 | 14.69 |
| I25 | 11 | 3.44 |
| I26 | 26 | 8.13 |
| I27 | 3 | 0.94 |
| I31 | 1 | 0.31 |
| I34 | 1 | 0.31 |
| I35 | 11 | 3.44 |
| I38 | 1 | 0.31 |
| I42 | 6 | 1.88 |
| I44 | 5 | 1.56 |
| I46 | 5 | 1.56 |
| I47 | 2 | 0.63 |
| I48 | 52 | 16.25 |
| I50 | 104 | 32.50 |
| I51 | 3 | 0.94 |
| J81 | 34 | 10.63 |
| Total | 320 | 100.00 |
| Asthma (≥ 18 years) | | |
| J45 | 228 | 97.85 |
| J46 | 5 | 2.15 |
| Total | 233 | 100.00 |
| Other ≥18 years (other primary ICD-10 categories) | | |
| A04, A09 | 4 | 0.24 |
| A27 | 1 | 0.06 |
| A40 | 2 | 0.12 |
| A40, A41 | 3 | 0.18 |
| A41 | 25 | 1.49 |
| A46 | 11 | 0.66 |
| A48, A49, B95, B96 | 23 | 1.37 |
| B34 | 4 | 0.24 |
| C32, C34, C50 | 5 | 0.30 |
| D46 | 1 | 0.06 |
| D50, D62, D64 | 14 | 0.84 |
| D69 | 1 | 0.06 |
| D84 | 2 | 0.12 |
| E10 | 1 | 0.06 |
| E11 | 1 | 0.06 |
| E16 | 1 | 0.06 |
| E64 | 1 | 0.06 |
| E83 | 1 | 0.06 |
| E86 | 5 | 0.30 |
| E87 | 21 | 1.25 |
| E88 | 1 | 0.06 |
| F05 | 4 | 0.24 |
| F10, F13 | 12 | 0.72 |
| F41 | 8 | 0.48 |
| F43 | 1 | 0.06 |
| F45 | 1 | 0.06 |
| F83 | 1 | 0.06 |
| G20 | 1 | 0.06 |
| G40-G41 | 1 | 0.06 |
| G45 | 2 | 0.12 |
| H34 | 1 | 0.06 |
| I10 | 3 | 0.18 |
| I11 | 6 | 0.36 |
| I12 | 1 | 0.06 |
| I60 | 1 | 0.06 |
| I63, I64 | 5 | 0.30 |
| I70 | 1 | 0.06 |
| I71 | 3 | 0.18 |
| I95 | 1 | 0.06 |
| J00, J01, J02, J03, J04, J06 | 18 | 1.08 |
| J31, J39 | 2 | 0.12 |
| J62 | 1 | 0.06 |
| J68, J70 | 2 | 0.12 |
| J69 | 16 | 0.96 |
| J84 | 11 | 0.66 |
| J85, J86 | 3 | 0.18 |
| J90, J91 | 22 | 1.31 |
| J93 | 25 | 1.49 |
| J95 | 2 | 0.12 |
| J96, J98 | 55 | 3.29 |
| K13 | 1 | 0.06 |
| K21, K22, K30 | 3 | 0.18 |
| K25, K26 | 3 | 0.18 |
| K40, K41, K42 | 4 | 0.24 |
| K52, K55, K57, K59, K63, K92 | 18 | 1.08 |
| K56 | 4 | 0.24 |
| K62 | 1 | 0.06 |
| K70 | 1 | 0.06 |
| K72 | 1 | 0.06 |
| K80 | 5 | 0.30 |
| K85 | 1 | 0.06 |
| L12 | 1 | 0.06 |
| L23, L50 | 13 | 0.78 |
| M10 | 1 | 0.06 |
| M31 | 1 | 0.06 |
| M54, M60; M62, M67, M70, M79 | 12 | 0.72 |
| M87 | 1 | 0.06 |
| N13 | 1 | 0.06 |
| N17, N18, N19, N20, N28, N39 | 27 | 1.61 |
| N30 | 19 | 1.14 |
| O82 | 1 | 0.06 |
| R00 | 2 | 0.12 |
| R04 | 4 | 0.24 |
| R05, R06 | 353 | 21.09 |
| R07, R10, R51 | 85 | 5.08 |
| R09 | 2 | 0.12 |
| R11 | 4 | 0.24 |
| R13 | 5 | 0.30 |
| R18 | 5 | 0.30 |
| R19 | 1 | 0.06 |
| R22 | 1 | 0.06 |
| R25 | 4 | 0.24 |
| R29 | 3 | 0.18 |
| R31 | 1 | 0.06 |
| R33 | 2 | 0.12 |
| R40, R41 | 5 | 0.30 |
| R42 | 2 | 0.12 |
| R49 | 1 | 0.06 |
| R50 | 17 | 1.02 |
| R52 | 3 | 0.18 |
| R53 | 25 | 1.49 |
| R55 | 10 | 0.60 |
| R56 | 4 | 0.24 |
| R57 | 4 | 0.24 |
| R60 | 3 | 0.18 |
| R63 | 1 | 0.06 |
| R67 | 5 | 0.30 |
| R79 | 3 | 0.18 |
| R91 | 1 | 0.06 |
| R99 | 3 | 0.18 |
| S00, S20 | 5 | 0.30 |
| S01 | 5 | 0.30 |
| S22, S32 | 3 | 0.18 |
| S27 | 1 | 0.06 |
| S42, S52, S72 | 6 | 0.36 |
| S51, S81 | 2 | 0.12 |
| S70, S80 | 2 | 0.12 |
| T06 | 1 | 0.06 |
| T14 | 1 | 0.06 |
| T17 | 4 | 0.24 |
| T18 | 1 | 0.06 |
| T38, T40, T42, T50 | 9 | 0.54 |
| T58 | 1 | 0.06 |
| T59 | 1 | 0.06 |
| T62 | 1 | 0.06 |
| T63 | 30 | 1.79 |
| T65 | 1 | 0.06 |
| T78, T79, T88 | 79 | 4.72 |
| Z01, Z03, Z04, Z51 | 494 | 29.51 |
| Z47 | 1 | 0.06 |
| Z74 | 2 | 0.12 |
| Z76 | 4 | 0.24 |
| Z87 | 1 | 0.06 |
| Z93 | 1 | 0.06 |
| Z94 | 1 | 0.06 |
| Z97 | 1 | 0.06 |
| Total | 1674 | 100.00 |
| <18 years | | |
| A40, A41 | 1 | 0.58 |
| B34 | 3 | 1.75 |
| E27 | 1 | 0.58 |
| F10, F13 | 1 | 0.58 |
| G40-G41 | 2 | 1.17 |
| J00 | 1 | 0.58 |
| J00, J01, J02, J03, J04, J06 | 12 | 7.02 |
| J05 | 25 | 14.62 |
| J12 | 2 | 1.17 |
| J13-J16 | 3 | 1.75 |
| J18 | 4 | 2.34 |
| J20 | 8 | 4.68 |
| J30 | 1 | 0.58 |
| J31, J39 | 1 | 0.58 |
| J44 | 2 | 1.17 |
| J45 | 74 | 43.27 |
| J46 | 4 | 2.34 |
| J96, J98 | 1 | 0.58 |
| R05, R06 | 8 | 4.68 |
| R07, R10, R51 | 3 | 1.75 |
| R56 | 5 | 2.92 |
| T17 | 1 | 0.58 |
| T63 | 1 | 0.58 |
| T78, T79, T88 | 3 | 1.75 |
| Z01, Z03, Z04, Z51 | 4 | 2.34 |
| Total | 171 | 100.00 |

Table S3: Patients classified as having AECOPD either had a primary diagnosis of COPD or a secondary diagnosis of COPD alongside a primary diagnosis that indicated a lung infection or displayed airway-related symptoms suggestive of a COPD exacerbation. A secondary diagnosis of COPD was defined within the ICD-10 categories J40-J44
